# Flexible IoT Agriculture Systems for Irrigation Control Based on Software Services

**DOI:** 10.3390/s22249999

**Published:** 2022-12-19

**Authors:** Eva Palomar-Cosín, Marisol García-Valls

**Affiliations:** Departamento de Comunicaciones, Universitat Politècnica de València, 46022 Valencia, Spain

**Keywords:** IoT software services, flexible sofware design, agriculture irrigation software, agriculture, software framework, alarm detection in IoT software

## Abstract

IoT technology applied to agriculture has produced a number of contributions in the recent years. Such solutions are, most of the time, fully tailored to a particular functional target and focus extensively on sensor-hardware development and customization. As a result, software-centered solutions for IoT system development are infrequent. This is not suitable, as the software is the bottleneck in modern computer systems, being the main source of performance loss, errors, and even cyber attacks. This paper takes a software-centric perspective to model and design IoT systems in a flexible manner. We contribute a software framework that supports the design of the IoT systems’ software based on software services in a client–server model with REST interactions; and it is exemplified on the domain of efficient irrigation in agriculture. We decompose the services’ design into the set of constituent functions and operations both at client and server sides. As a result, we provide a simple and novel view on the design of IoT systems in agriculture from a sofware perspective: we contribute simple design structure based on the identification of the front-end software services, their internal software functions and operations, and their interconnections as software services. We have implemented the software framework on an IoT irrigation use case that monitors the conditions of the field and processes the sampled data, detecting alarms when needed. We demonstrate that the temporal overhead of our solution is bounded and suitable for the target domain, reaching a response time of roughly 11 s for bursts of 3000 requests.

## 1. Introduction

One of the resources that is most affected by climate change is fresh water, which is 3% of the whole amount of water in the planet. This includes frozen water: glaciers and polar ice caps. As such, available fresh water is a small percentage and a precious resource.

Activities that consume the most fresh water, such as the irrigation of crops and massive recreational centers (among others), have to be carefully managed for extreme efficiency. Precisely, irrigation is the activity that consumes the most, being aproximately 70% of the overall quantity [1,2]. Not long ago, the visual inspection for decision making on the irrigation of crops was common practice. According to [3], this was the case in around 80% of US farms.

During the last decade, IoT technology has successfully been adopted in the vast majority of application domains, including agriculture [4] as a basic instrument for implementing a resource-efficient operation. As a result, the IoT paradigm is a key step in the way that modern systems are conceived and architected. The interconnection of a miriad of sensing and actuating nodes constitutes a powerful infrastructure to develop applications that monitor and actuate on the physical objects and environment, enhancing the intelligence and capacities of the human operators and users. The application areas of IoT span from eHealth [5], to smart manufacturing [6] and automation, smart cities, and many more. IoT technology applied to agriculture irrigation can significantly contribute to the rational usage of water as abundant and precise data are obtained from the field in real-time. This serves greatly to improving the decision making on irrigation periods and quantities, among others.

In the last decade, the number of contributions that apply the IoT technology to agriculture has increased significantly. The vast majority of contributions provide a system-level architecture that monitors different parameters such as characteristics of the soil and the environment; these data are fed some target solution platform tailored to the particular needs of individual agriculture fields. Other solutions typically target the design of the hardware, i.e., the sensors that will be later located on the physical deployment. However, to the best of our knowledge, there are no software-centric solutions for the development of IoT software systems for agriculture. The few solutions where the software side is present are reduced to a pure instrumental level, such as the usage of a particular API for information exchange among the deployed computer-nodes (IoT sensors, single-board computers, etc.) and deployed nodes and the cloud backends. There is a gap at the level of software-centered solutions that support the flexible modeling and development of IoT agriculture.

This work designs and prototypes a software framework for actively monitoring the water management applied to irrigation in agriculture. We propose a software framework that can extend the capacities of mainstream IoT platforms by providing a strategy to design IoT agriculture software in a flexible manner. Our framework is based on software services that model the front-end functionality of the interacting nodes in IoT systems that use a client–server communication scheme. The system model is based on federated areas with controlling servers that: (i) store monitored parameters, (ii) analyse the data to detect alarms that may generate corresponding actuation values; and (iii) send the actuation values to the IoT devices. A decomposition of the software services into their constituent operations and functions is also contributed both for client and server sides; and the mapping of these to the main functionality such as detection and actuation in front of anomalies and alarms is provided. We prototype the software framework for an irrigation control IoT software system and demonstrate that the interactions are achieved efficiently with a maximum time of 11 ms for the worst-case situation.

The paper is organized as follows. Section 2 elaborates the target application scenario, analyses the possible communication strategies that can be used, and the common functionality of mainstream IoT platforms. Section 3 describes the approach with the design process based on software services; the decomposition of the internal structure of services; and the proposed framework that details its constituent software modules, including the alarm detection module. This section includes the mapping of the internal software services to the framework modules. Section 4 describes the prototype implementation of the proposed framework applied in the context of the IoT software system for controlling irrigation in agriculture. It defines the IoT sensoring infrastructure and associated servers, as well as the alarm conditions that guide the irrigation control. A number of experiments are also presented that validate the temporal behavior of the designed framework. Section 5 presents the related work. Section 6 concludes the paper.

## 2. Application Model

### 2.1. Scenario Overview

The target scenario is an IoT agriculture systems that provides eco irrigation. The IoT substrate is composed of smart sensors that sample soil and environment data; and the server side that gathers and processes the sampled data. For this purpose, a software framework is proposed on the IoT substrate to achieve eco irrigation: The plants receive the needed water, but the software platform controls the quantity, amount, and frequency of irrigation based on the data sampled by smart IoT devices located in the field.

The system includes a server platform based on services technology. Moreover, the software functions of IoT sensor devices are also service-based.

One of the goals of the designed platform is that the temporal behavior of the software logic running on the IoT devices is appropriate to achieve real-time monitoring, detecting possible anomalies or alarms, and actuating as needed.

**Figure 1:**
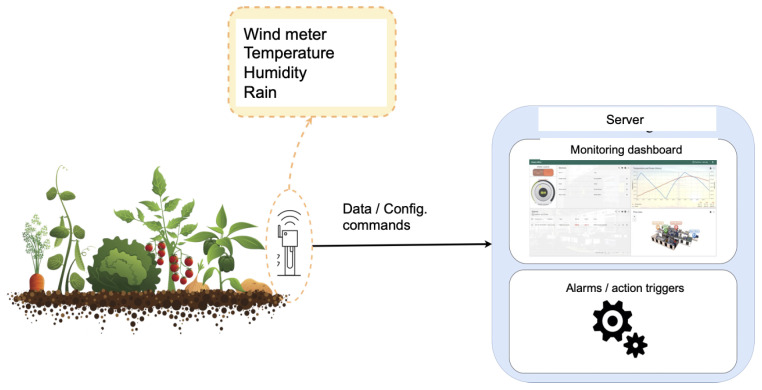
System overview.

Figure 1 illustrates the target system that includes sensors to report real-time readings of wind speed, rain quantity, humidity, and temperature. Sensors are connected to the control server by means of a well-defined interface. The server receives real-time data; then, it processes these data, and generates configuration commands back to the IoT nodes.

The integrated logic analyses the effects of the current atmospheric conditions such as temperature, wind, and humidity, to activate the irrigation system. This logic also considers the potential rain, as it includes a pluviometry sensor.

### 2.2. Communication Options

There are different options to implement such a scenario as far as the communication platform is concerned and the integrated software libraries. If bare RPC (*Remote Procedure Call*) software is used, faster exchange rates are achieved at the cost of supporting only one-to-one communication between client and server sides. Using P/S (*Publish-Subscribe*) supports multiple interaction models (1 to many, many to many, etc.) but the needed distribution protocols to ensure coherency and non-duplication introduce higher overheads that may not pay off in many IoT scenarios. Moreover, implementing this scenario with a communication protocol such as pub-sub or RPC is possible but error prone and lacks sufficient flexibility to accomodate new nodes and functionality, which only need to communicate to an area server.

Using REST-based interactions (such as [7]) to design and deploy this scenario enhances flexibility; but it comes at a cost that is the introduction of the protocol headers and the associated cost of parsing them in the higher levels of the protocol. However, this can be aleviated by using web-like interactions for constrained environments such as the CoAP [8] protocol (*Constrained Application Protocol*). Additionally, the client software (browser) is more time consuming than a native C software directly run on the hardware and operating system. However, the lack of support functions of the native C software modules will have to be crafted manually.

### 2.3. Mainstream IoT Platforms

**Figure 2:**
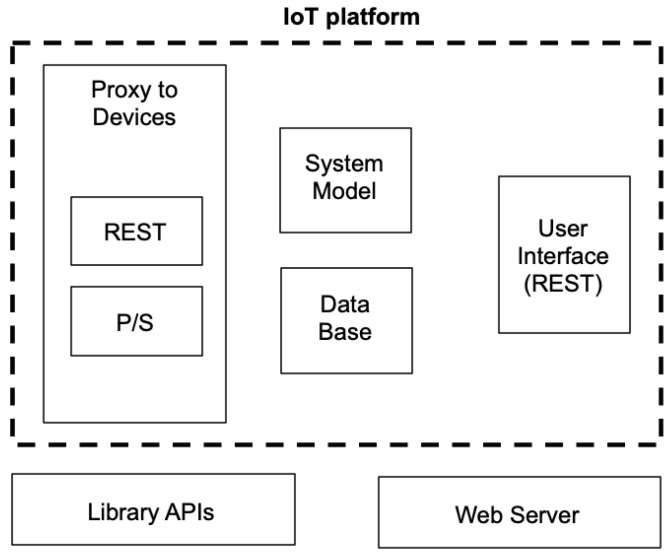
The structure of IoT platforms.

Most current IoT platforms have a set of common modules as part of their structure. The commom part is shown in Figure 2. The IoT platform boundaries are expressed with a dashed line.

There are four main components that provide the basic harness and functions. These are explained below.

The interface to IoT devices (contained inside the *Proxy to Devices* block) is performed essentially by means of two different protocols: REST [9] (*REpresentational State Transfer*) or publish–subscribe. There are a number of particular instantiations and libraries that realize both of them. Interaction through REST provides a pure client–service model, and it improves compatibility with web protocols; whereas interaction by means of publish–subscribe models constitute a powerful *n* to *m* communication scheme based on a consumer–producer model.

In the vast majority of current platforms, the interface to external users (such as system operators and other actors that only visualize data) is performed by means of a web-based protocol and a REST communication scheme. This function is represented with the *User Interface (REST)* block. The usage of the web is due to the natural penetration of web technology in virtually all application domains. The popularity of web protocols is due to a number of reasons: its simple communication model; its ease of use; the improvement of the performance of current tools; powerful visualization capacities; and natural integration with paradigms such as cloud computing backends.

Additionally, most IoT platforms have a built-in block that stores the system information (the *System Model* block). It provides facilities to define the devices and nodes that are part of the system, their characteristics and interaction links. This block uses a data base software (expressed here as *Data Base* block) that persistently stores the data of the system: both the system model and the data gathered and generated by the platform itself.

Logic such as artificial intelligence and machine learning functions usually relies on employing external libraries (library APIs), as it is typically not part of the internal core per se.

## 3. Approach

This section describes the proposed software framework that enhances the functionality of common IoT platform functions. The target IoT infrastructure is specified as a set of software services; we understand the concept of *software service* as a self contained functionality piece that exposes a well-defined interface through which it receives and transmits data.

Firstly, the service-oriented system model is explained that provides a simple interaction scheme among system nodes. Then, we explain the proposed framework, describing the modules that it integrates.

### 3.1. IoT System Model Based on Services

**Figure 3:**
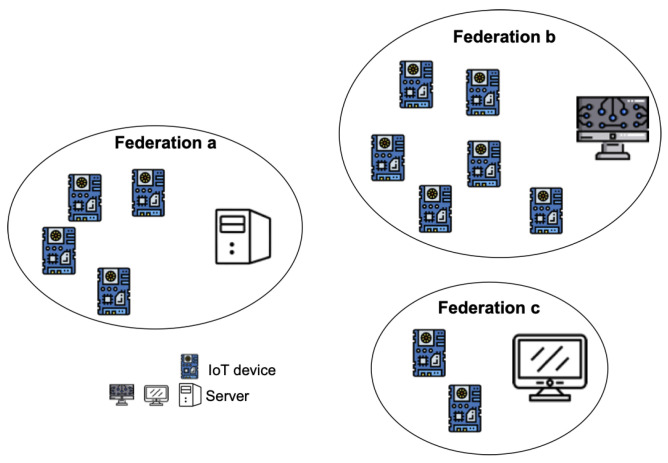
IoT system federations.

The target IoT system is based on the existence of federations of nodes (see Figure 3) that include a number of smart IoT devices and an associated server. Federations have been applied extensively in distributed systems and in more recent computing paradigms such as fog [5]. One of the reasons is the inherent ordering that favors controllability and accountability of nodes; this is particularly useful in the case of errors or missfunctions, as it becomes simpler to trace back until reaching the fault source.

In our approach, the functionality offered by nodes is modeled as *services* and the interactions across nodes are modeled as service invocations and/or requests. This means that all nodes involved in the IoT system (e.g., smart IoT devices and server nodes) expose their functionality as service APIs (*Application Programming Interface*) over a REST [9] communication scheme. In this scenario, IoT devices monitor and actuate on the physical environment. Monitoring implies gathering data about the field conditions and sending these data to the server; whereas actuating implies receiving actuation commands from the server to execute them on the physical system. The server controls the system actuation. This means that the server configures the smart IoT devices operation, and stores the sampled data.

**Figure 4:**
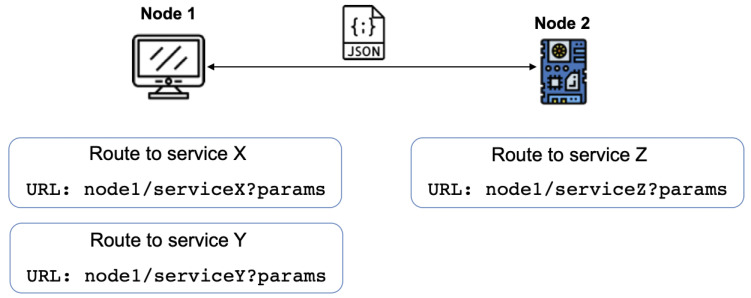
Service-based communication scheme.

Figure 4 illustrates the interaction model across the nodes involved in our target IoT system. A node can request information from (or send information to) another node by using the appropriate REST method. The communication invocation is performed through a well-formed URL (Uniform Resource Locator) that includes:

- node part, representing the protocol and the node domain.
- serviceX part, that includes the path to service *x* inside the server, and
- params part, containing the associated parameters that are the precise details about the request.

The specified parameters follow the scheme of URLs; this means that requests can contain none or multiple parameters separated by the ‘&’ character in the form of key-value pairs. An example could be: temp=24&wind=12. For the sake of clarity, Figure 4 shows an explicit URL containing all parameters. However, it is important to clarify that the parameters (the key-value pairs) are communicated in a post method in a REST scheme. That is, exchanged data are not communicated in a visible manner, but as part of the message body.

Messages exchanged among nodes follow a format well suited to generic services. The framework uses a de facto standard such as JSON [10] (*JavaScript Object Notation*) for data exchange. JSON is nowadays one of the most widely used formats for text-based data exchange in the majority of domains and especially in web services and REST-based interactions.

A server-based design approach brings in a number of benefits. Firstly, it provides the needed combination of expressive power, showing the connections and interelations of clients and server sides; also, it offers the needed control over the communication, since such scenarios employ a REST-based interaction that is a plain client–server scheme. Additionally, this scheme translates into a resource efficient communication that can be achieved by using the communication protocols for constrained environments.

**Figure 5:**
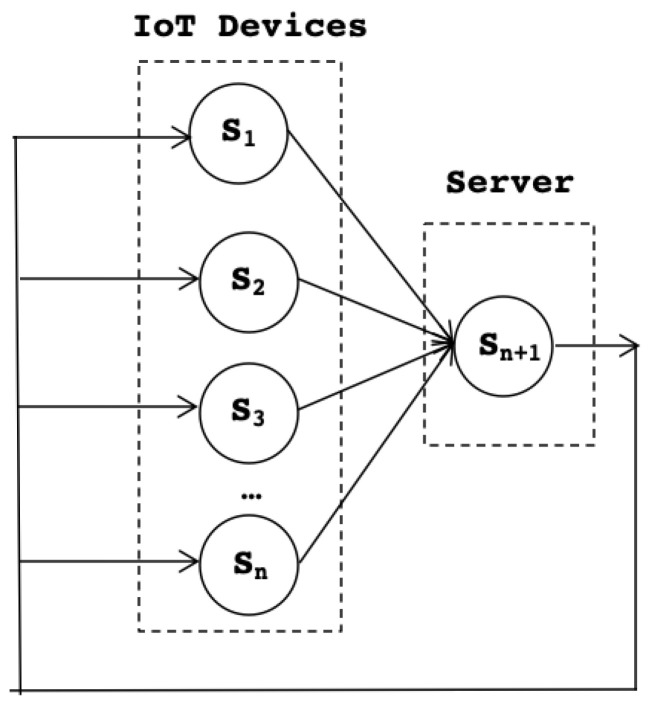
Service scheme at server and IoT devices.

Figure 5 illustrates the service-based definition with *n* smart IoT devices that are connected to a server. At the top level, Figure 5 shows the service in the system. This sample model shows a simplified version containing only one service per IoT node. The model easily scales to more complex service configurations.

**Figure 6:**
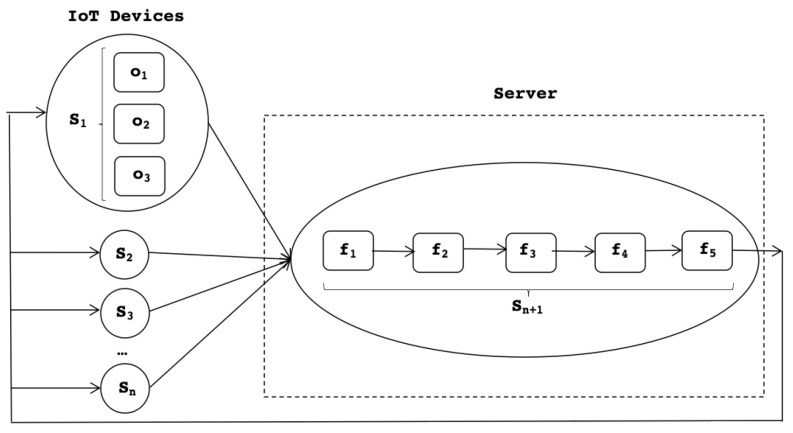
Expanded services scheme.

This big picture is then further expanded in Figure 6 that shows how each service is also decomposed into a number of activities that collaborate to provide an integrated functionality towards the outside nodes. To differentiate, constituent activities of IoT devices are named *operations*; and constituent activities of server nodes are called *functions*. At the server side, the constituent functions are assembled in a pipeline scheme, as the data produced by each operation are fed to the subsequent one. Inside an IoT node, the operations of a given service are executed in parallel, as explained below.

There are *n* IoT devices. Each IoT device has a set of services: $s_{1},\cdots s_{n}$. The server has one software service, namely $s_{n+1}$, that includes the logic for managing the *n* IoT devices. Figure 6 shows the decomposition of services of IoT devices such as service $s_{1}$ that is composed of three operations $o_{1},o_{2},o_{3}$; such operations can carry out actions such as updating the sample rate of the device ($o_{1}$), issuing actuation commands on the actuator part of the IoT device ($o_{2}$); and performing self-healing and self-monitoring actions that result in sending some log data to the server ($o_{3}$).

At the server side, service $s_{n+1}$ is made of the server functions $f_{1},\cdots f_{5}$. This means that the server side functionality contains a pipeline of four services: $f_{1}$ is the entry module acting as a gateway and receiver for the incoming data from the remote smart devices; $f_{2}$ is the parsing function that recognizes the incoming data and language, and rebuilds the received elements and code entities; $f_{3}$ is the rendering logic that presents each received element on the IoT system dashboard; $f_{4}$ is the analysis functionality that determines the response to IoT devices, i.e., further (if any) configuration commands to be sent to the smart devices for either actuation or self-tunning (e.g., adaptation of sampling period, etc.); $f_{5}$ is in charge of submitting the response to the IoT devices.

IoT device services represent the physical devices that can be invoked via either a client–server or a pub–sub protocol. The result of the invocation will be a resource specified in JSON format, e.g., {“temperature”: 15} for some temperature sample readings.

IoT devices provide the sample readings to the IoT system platform using web-based communication and the rest paradigms with a post request such as https://www.serverabc.com/api/iotsys16543/telemetry that invokes a particular service to which it passes a type Content-Type: application/json for the associated data {“temperature”: 15}.

### 3.2. Software Modules Overview

**Figure 7:**
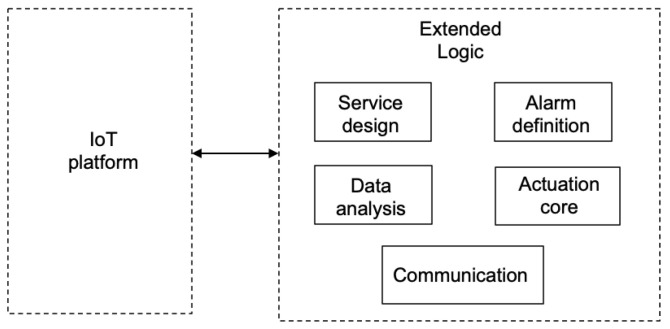
Proposed solution and enhancement.

The software framework is architected as shown in Figure 7, containing a set of modules that extend the common functions of IoT platforms in the various ways listed below.

- The IoT system needs to be architected based on services as decoupled functionality pieces that expose clean interfaces. This is supported by the *service design* module. A service-based design is highly flexible and naturally supports scalability in the physical scenario. Adding or removing an IoT device can be mapped to the presence or absence of a software service at the logical level.
- Alarms and actuation logic programming using event-based technology is suppported by the *alarm definition* module. This part provides an external enhancement to the identification and detection of alarms, the specification of new actuation conditions, or the addition of threshold value checking. The module supports the programming of these functions in an event-based language to ensure maximum web compatibility, as most platforms expose web interfaces and use web communication protocols.
- Support for data analytics. More complex logic can be programmed for guiding the actuation indications of the server on the field IoT devices. This is supported by the *data analysis* module.
- The *actuation core* module is the central point of the framework operation. It guides the actuation based on the inputs from the modules alarms and data analytics. It leverages the communications module to interact with the devices.
- A *communication* module is provided that implements the mapping to the particular networking protocols that need be used to properly interact with IoT devices and users.

**Figure 8:**
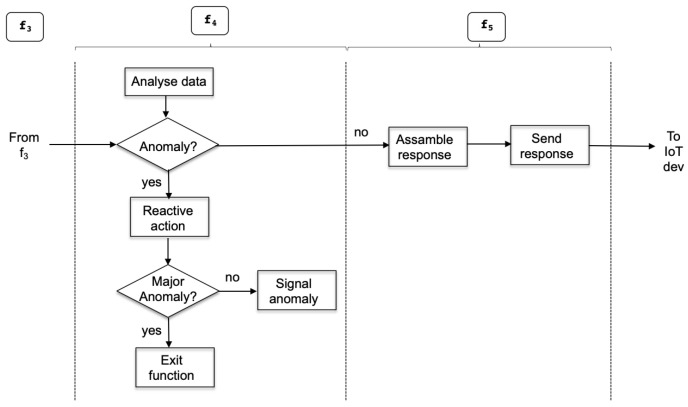
Anomaly/alarm detection and associated activities.

The analysis function $f_{4}$ runs the logic to detect the occurrence of situations representing an anomaly. This may trigger the system to perform corrective actions or to react to mitigate the foreseen effects. These ocurrences are named *alarms* and they are programmed inside function $f_{4}$ in an event-based mode; precisely, they are part of the *alarm definition* module. Figure 8 shows the activities that take place inside the server functions related to the detection of an anomaly or alarm. Detected anomalies may have two different consequences. Sometimes, they may only require to be signaled to the operator (e.g., display a value highlighted in some red color to inform of some exceptional value or situation); however, it may be the case that the alarm implies some catastrophic effect for the system and this may require one to invoke some exit function that discontinues the system operation and requests the operator control until the situation is solved.

Mapping the server functions to the designed software module is straightforward. On the one side, function $f_{4}$ includes modules *actuation core*, *data analysis*, and *alarm definition*. The *actuation core* module is a leading and central part, as it determines the execution flow shown under $f_{4}$ of Figure 8. On the other side, function $f_{5}$ includes the *communication* module to interact with external actors in the needed underlying networking protocols.

### 3.3. Alarm Definition

Alarms express the occurrence of an event or entering a situation that requires that an urgent action is taken. Examples of such actions are turning on a red light to indicate danger, and some physical action to avoid negative consequences.

In our solution, an alarm is programmed using conditional language and boolean expressions. An example of supported alarms are explained in what follows.

**Definition 1.**  *A* ***single variable alarm*** *is an expression of type* *if* *e* *then action1* *, where e is an expression of type v⊕δ that involves checking the value of a variable v against a prefixed threshold δ. The symbol ⊕ represents an operation of types =, <≤, >, ≥, so the expression is evaluated to either true or false. If the evaluation result is* *true* *, then* *action1* *is performed. The alarm can be extended with an* *else* *clause that will be executed if the expression is evaluated to false, yielding the execution of* *action2* *. An example of a single variable alarm is* *if* *temp<15.5* *then flash red led* *, temp being a variable that holds temperature variables.*

*A* ***multi-variable alarm*** *is an expression of type* *if* *e* *then action1* *, where e can be decomposed into multiple (at least two) binary expressions such as $e_{1}⊕e_{2}$, where $e_{1}$ and $e_{2}$ are expressions that may contain multiple variables, logic operations, or scalar values. ⊗ represents logical operations such as logical AND, OR, NOT, among others. Expression e and all its subexpressions $e_{1}$, $e_{2}$, etc., evaluate to either true or false. As in the previous case of single variable alarm, the specification can be extended with an* *else* *clause that will be run if the expression is evaluated to be false.*

## 4. Implementation and Results

Our solution is applied to the development of an eco irrigation system in the IoT agriculture for delivering a solution that is capable of flexibly modifying the irrigation conditions and alarms to experiment with implementation options in search for the maximum benefit to the agriculture ecosystem and for water saving.

In this case, the design of the system contains four smart IoT devices controling four respective sensors to measure wind, temperature, humidity of the environment, and rain conditions. These devices are connected to an IoT platform [11] with capabilities of basic device connectivity and telemetry presentation to which our approach is integrated.

The above provided modeling is instantiated in what follows. The client side services are $s_{1},\cdots ,s_{4}$, with each associated to the previously mentioned IoT devices; and $s_{5}$ is the server-side service. Each client-side service runs operations $o_{1},o_{2},o_{3}$. For the case of the wind sensor, $o_{1}$ corresponds to setting the sampling frequency of the wind speed or wind direction at a software level; $o_{2}$ corresponds to further actuation of the device on itself, e.g., reducing the wind vane frequency if the wind direction is oscillating; and $o_{3}$ corresponds to setting the logging mechanisms of this sensor to report its performance.

For the alarm definition, the scenario identifies a set of operation conditions that determine whether the system operation is normal; or determines whether an alarm has been fired that requires action or simply informing the operator. There are single and multi-variable conditions:

1. Wind effect. The wind is one of the worst enemies of irrigation, as it has a decisive influence on the distribution and uniformity of the sprinkled water. This problem is aggravated by the speed and direction of the wind. In the following are some recommendations for a particular field and crops:
  - For wind speeds over 8 km/h, it is recommended to decrease the sprinkler spacing by 2% every 1.5 km/h increase in wind speed.
  - For winds speeds exceeding 20 km/h, it is recommended not to irrigate; thus, pumps must be stopped.
2. Temperature. Each crop has its own optimum temperature to reach the highest yields. If the plant temperature rises above the optimal point, precision irrigation is automatically activated. In the following are some recommendations for a particular field and crops:
  - Acceptable temperature values must stay in between 23 °C and 32 °C. Fruit quality drops outside this range.
3. Humidity. Acceptable ambient humidity is related to the particular plants. Moreover, the sensibility to water excess or deficit is also dependent on the particular plant and soil quality (e.g., carbon presence in soil). Such data are specific for each type of soil and are related to the humidity value at a matrix potential. The condition to be checked here is shown below:
  - A humidity value in the range from −20 kPa to −75 kPa is considered normal.
4. Pluviometry. It is used in agriculture for irrigation control. This agricultural sensor records and sends data about the additional water content or the amount of precipitation. The value of the pluviometry sensor may cause the irrigation pump to be disconnected. A recommendation considered in this example is that values less than 50 L in a week are considered normal and do not have an effect in the irrigation process. Values over 100 L will stop the irrigation pump for a day.

Table 1 shows the conditions checked in the system in order to decide whether the irrigation process has to be adjusted. The first column represents the normal operation state; in this state, any action on the irrigation pump is not required, so that the irrigation process will continue to operate normally. The second column determines a moderate risk on the effectiveness of the irrigation due to windy conditions, mild temperatures, or increasing humidity; this will require one to decrease the irrigation level, avoiding unnecessary watering. The third column identifies risky conditions due to, for instance, high winds; this will result in stopping the irrigation process, as it will have a negligible effect on the actual watering of the crops.

In the following, it is demonstrated how the alarms are implemented in the software framework that reflect the conditions specified in Table 1. The algorithm is shown in JavaScript, which is the language in which the alarm detection module is implemented.

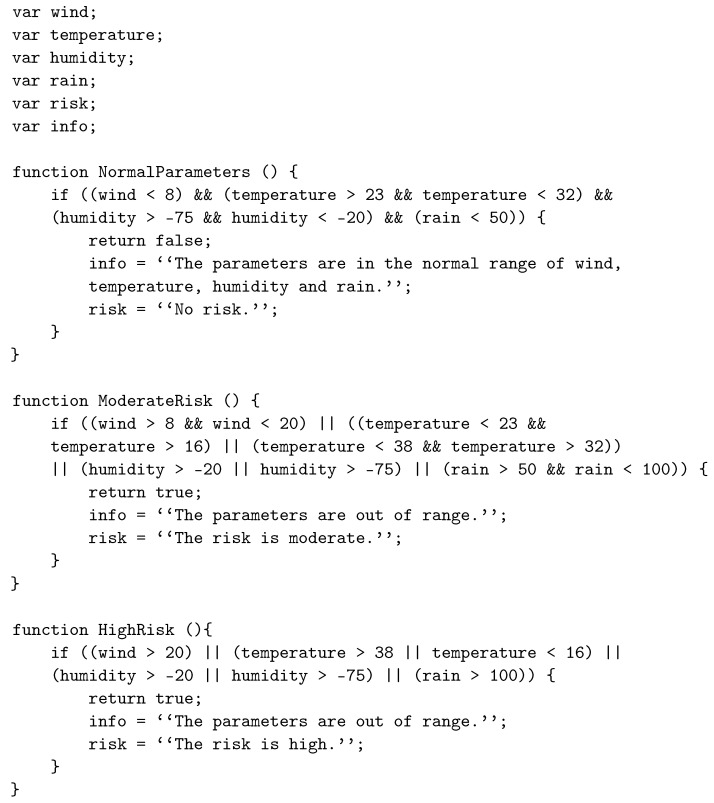



The software framework has been tested to ensure that the proposal conforms to the needed temporal behavior. We have used the bare capacities of an IoT platform [11]. As a persistent storage option, we have used a PosgreSQL database. Our interest is to validate our solution, obtaining measures about the response time perceived by the client side. Then, we have measured the communication interaction at the client side, detecting the time when the server initiates the sending of the response. Table 2 shows this response time behavior for different request bursts and for four different experiments (E1 through E4). We observe that for a light demand from the framework (i.e., a burst of 10 service requests), the cost is 229 ms. A mid-low burst rate leads to a cost of 1.02 s; whereas, the communication becomes more efficient for high (1000) and very high (3000) bursts with a cost of 2.31 s, and 9.349 s, respectively. Moreover, we have obtained the average times for each burst rate.

Table 3 shows the average cost per request. It is observed that, as the number of requests increases, the time it takes for each request to reach the server and back decreases. The observed trend is the one that was initially expected. That is, for a high request number, the underlying platform is close to its saturation; and it saturates if we cross the limits with a very high request burst-rate.

**Figure 9:**
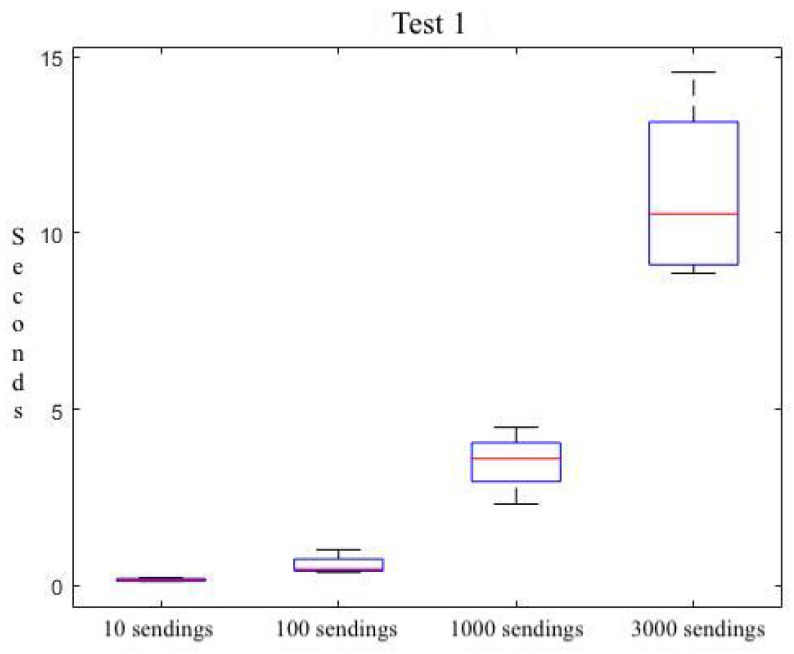
Summary of temporal behavior of test 1.

The trend can be better compared with the plot shown in Figure 9. Here, a summary of all the collected data is shown in four descriptive measures. The size of each box marks the dimensions of the IQR (*InterQuartile Range*): the difference between the third and first quartiles of the distribution. The median value is marked with a red line inside each box. Minimum and maximum values are represented by short horizontal lines outside of the box. If any outliers are present, they are marked as red crosses.

Additionally, we have designed and performed a second test on the temporal behaviour of our solution for the communication of different data sizes. It is important to recall that in the first test, we have explored the temporal behavior of our solution and have explored the limits and utilization boundaries of the underlying IoT platform. Then, this second test stays within the boundaries that allow the underlying IoT platform to function normally, as derived from the initial test.

In the second test, we have used the JSON data exchange format (also used for serialization) and explored the temporal cost for different burst rates (*low* for 10; *medium* for 100; *high* for 1000; and *very high* for 2000). Table 4 shows the obtained results, as we progressively vary the number of requests and the size of the exchanged data.

Analyzing Table 4, we observe that increasing the size of the JSON file does not increase the arrival time of the data. For instance, focusing on the temporal cost for a low burst-rate, we observe a similar cost for all data sizes (13 bytes through 3312 bytes) with 225 μs difference. On the other hand, we observe a behavior that is coherent with the initial test: increasing the request burst-rate does not directly impact the communication time.

**Figure 10:**
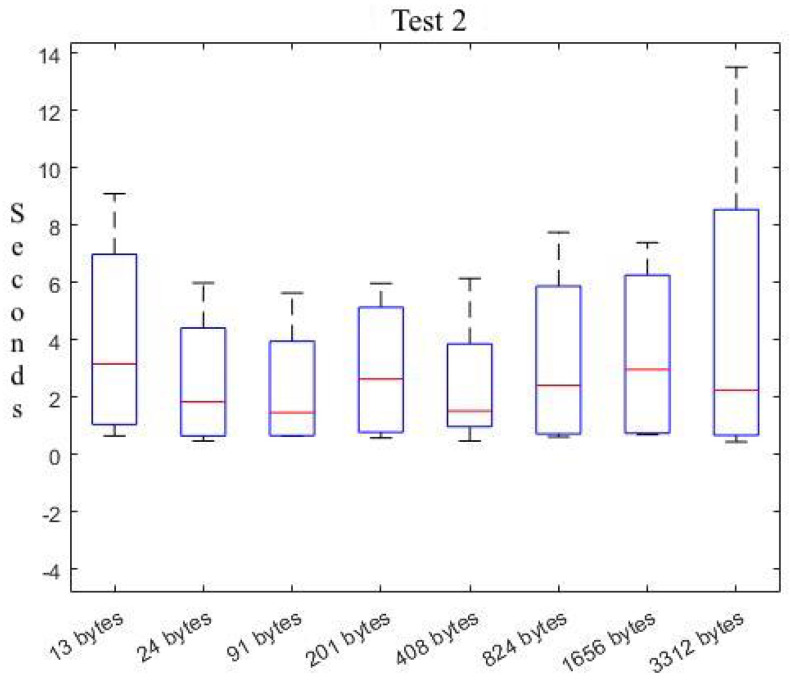
Summary of response times.

Figure 10 summarises the results of the test, exhibiting a quite stable lower bound of the first quartile temporal results for all data sizes. There are evident differences between the upperbound, but these are due to the internal characteristics of underlying platfom.

In summary, the effect of the proposed solution is minimal and the temporal behavior is directly related to the underneath layers that we analyzed in the initial test. We observed that the sending time increases as the number of requests raises. This was evidenced in the accumulated times; but also when breaking down the total into average cost, as the latter decreases with the number of request increases. These tests allow us to extract conclusions such as, for example, what to do to reduce the sending time. For this, smart IoT devices that do not require real-time interaction can be programmed to collect data from a number of sampling periods and summarize them in a single JSON data message. However, we must consider the downside, which is that the cost of sending small JSON objects is higher given the inherent productivity of the underlying communication protocol such as [12], among others.

## 5. Related Work

IoT technology applied to agriculture irrigation can significantly contribute to the rational usage of water, as abundant and precise data are obtained from the field in real-time.

A number of works have appeared for IoT agriculture irrigation over the last decade, right when the IoT paradigm experimentation became popular. It is important to note that the literature related to the IoT agriculture system experiments has a large bias towards sensor-hardware development and final system development that achieves the initially specified functional characteristics. This is evidenced in [13] and particular examples are are [14,15,16]. Nevertheless, to the best of our knowledge, there are practically no contributions that take a software-centric perspective to IoT agriculture. The software is typically taken as an instrumental piece in the context of an overall final system development. This is a common ground to other IoT domains such as medical systems or industrial automation and manufacturing [17,18].

Approaches such as [19,20] focus on the design of customized sensors for irrigation and sensing of field characteristics. These are different from our approach in that they focus on the hardware design of the IoT sensors.

Other research lines have engineered solutions for the automated irrigation in fields focusing on the system level view such as [21,22]. Some contributions such as [23] focus on the hardware and also provide the design and development of an operator interface for usability, but not focusing on interoperability. They are different from ours in that they target a particular deployment; whereas, we propose a rather software-oriented approach to design the system based on the web service interfaces that the intervening nodes (IoT sensors and servers) provide. This provides high interoperability and yields to modular designs of IoT systems, where nodes can be easily replaceable.

Other works have combined wireless sensing, IoT technology, and the cloud for storing the monitored data. Examples are [24], which uses the cloud to store the sensed data; Ref. [25], which provides a study on the actual performance of communication between wireless sensing and the cloud backend; Ref. [26] relies on the inherent usage of services for monitoring without further attention and uses the cloud backend to store monitored parameters on which to later process to infer the actuation actions; others such as [27] that have approached the fog paradigm in the search for improving performace, although it is a quite immature contribution; or [28], which designs a smart irrigation system that stores the monitored field values in the cloud. However, these cloud-based approaches relying on the storage of data in the cloud servers can be improved by using storage closer to the physical object (the field), as we propose. Additionally, our solution provides a structured service-based design scheme that allows designers and engineers to draw an initial map of the application-level logic that will be later more easily instantiated in the physical domain.

Intelligent decision making in combination with the IoT sensing of crops has also been explored in a number of solutions. This is in the case of [29] for improving temperature management in greenhouses, which has an effect in irrigation; Ref. [30] that provided an initial tracing pattern over business models based on events; Ref. [31] that reviews a number of other contributions on agricultural IoT technology in general to which artificial intelligence is also applied; or [32], which proposes a neural network used for decision making respecting water supply, and fertilizer spray, among others.

There are also a number of contributions on system-level architectures for IoT-based agriculture that directly or indirectly impact water management. In [33], it is presented how to model solutions for monitoring water content in the soil. Another example is the contribution of [34], which describes in detail the most commonly required components (mostly focusing on hardware) of an IoT-based irrigation system.

As a result, and to the best of our knowledge, there is no solution that contributes a software framework to design IoT agriculture irrigation systems from the point of view of architecting them flexibly based on software services. This software-centric approach provides a modular scheme that can be integrated in current IoT platforms, preserving the temporal behavior required by the system. We use our previous work [35] describing an evaluation process for IoT platforms to perform the temporal validation of our proposal. This previous work provides a structured baseline to identify the relevant software aspects in an IoT project in order to validate the temporal behavior of key software pieces of the overall system such as the IoT platform.

## 6. Conclusions

This paper has proposed a software framework that integrates strategies to design and develop the software of IoT systems for irrigation control. The approach allows designers to easily model IoT systems based on software services that interact through REST-based protocols over a client–server model. The framework decomposes the client and server sides of an IoT system into the precise constituent functions and operations, to draw a clean map to the designer of where to hook further software services that may be needed to enhance the overall functionality of the system. As a result, the framework enhances the capacities of mainstream IoT platforms, providing a software hook that supports the programming of the alarm detection to improve the decisions over irrigation instants. Experiments have demonstrated that the temporal behavior of the proposed framework and communication between client and server preserves the required latencies and validates the solution. We show that even for high communication bursts of 3000 requests, the latency is kept consistently below 11.2 s.

## Figures and Tables

**Table 1:** Definition of risk levels for alarm detection.

No Risk	Moderate Risk	High Risk
wind < 8 km/h	wind > 8 km/h AND wind < 20 km/h	wind > 20 km/h
temperature > 23	(temperature < 23 AND temperature > 16)	temperature > 38
AND	OR	OR
temperature < 32	(temperature < 38 AND temperature > 32)	temperature < 16
humidity > −75	humidity > −20	humidity > −20
AND	OR	OR
humidity < −20	humidity > −75	humidity > −75
rain < 50	rain > 50 AND rain < 100	rain > 100

**Table 2:** Response time in front of different request rates.

Request Burst	E1	E2	E3	E4
10	0.229403	0.164141	0.129209	0.125360
100	1.020914	0.383103	0.493095	0.448629
1000	2.316834	3.6164326	4.487297	3.591282
3000	9.349804	8.853687	11.730795	14.558191

**Table 3:** Average response time (in ms) in front of different request rates.

Request Burst	Average
100	5.864345
1000	3.502961
3000	11.123119

**Table 4:** Temporal behaviour as the exchanged data size and the request bursts increase (in seconds).

Data Size (In Bytes)	Low	Medium-Low	High	Very High
13 bytes	0.645241	1.444396	4.866774	9.102204
24 bytes	0.831007	0.462578	2.845448 s	5.985796
91 bytes	0.640276	0.659791	2.258518 s	5.636820
201 bytes	0.968613	0.573597	4.297246 s	5.968502
408 bytes	0.468909	1.569857	1.457807 s	6.145699
824 bytes	0.828033	0.606376	3.987770 s	7.754083
1656 bytes	0.794128	0.687427	5.127063 s	7.394538
3312 bytes	0.894942	0.438648	3.574610 s	13.520767

## Data Availability

Not Applicable.
